# Kinase Inhibition Leads to Hormesis in a Dual Phosphorylation-Dephosphorylation Cycle

**DOI:** 10.1371/journal.pcbi.1005216

**Published:** 2016-11-29

**Authors:** Peter Rashkov, Ian P. Barrett, Robert E. Beardmore, Claus Bendtsen, Ivana Gudelj

**Affiliations:** 1 School of Biosciences, University of Exeter, Exeter, United Kingdom; 2 Discovery Sciences, Innovative Medicines and Early Development, AstraZeneca, Cambridge, United Kingdom; Massey University, NEW ZEALAND

## Abstract

Many antimicrobial and anti-tumour drugs elicit hormetic responses characterised by low-dose stimulation and high-dose inhibition. While this can have profound consequences for human health, with low drug concentrations actually stimulating pathogen or tumour growth, the mechanistic understanding behind such responses is still lacking. We propose a novel, simple but general mechanism that could give rise to hormesis in systems where an inhibitor acts on an enzyme. At its core is one of the basic building blocks in intracellular signalling, the dual phosphorylation-dephosphorylation motif, found in diverse regulatory processes including control of cell proliferation and programmed cell death. Our analytically-derived conditions for observing hormesis provide clues as to why this mechanism has not been previously identified. Current mathematical models regularly make simplifying assumptions that lack empirical support but inadvertently preclude the observation of hormesis. In addition, due to the inherent population heterogeneities, the presence of hormesis is likely to be masked in empirical population-level studies. Therefore, examining hormetic responses at single-cell level coupled with improved mathematical models could substantially enhance detection and mechanistic understanding of hormesis.

## Author Summary

Hormesis is a highly controversial and poorly understood phenomenon. It describes the idea that an inhibitor molecule, like an anti-cancer or anti-microbial drug, can inadvertently stimulate cell growth instead of suppressing it. This can have a profound effect on human health leading to failures in clinical treatments. Therefore, getting at the mechanistic basis of hormesis is critical for drug development and clinical practice, however molecular mechanisms underpinning hormesis remain poorly understood. In this paper we use a mathematical model to propose a simple and yet general mechanism that could explain why we find hormesis so widely in living systems. In particular, we discover that hormesis is present within a fundamental structure that forms a basic building block of many intracellular signalling pathways found in diverse processes including control of cell reproduction and programmed cell death. The benefits of our study are two-fold. Having simple molecular understanding of the causes of hormetic responses can greatly improve the design of new drug compounds that avoid such responses. Moreover, due to the fundamental nature of the newly proposed mechanism, our findings have a potential broad applicability to both anti-cancer and anti-microbial drugs.

## Introduction

Hormesis is a phenomenon describing biphasic dose response relationships that exhibit low-dose stimulation and high-dose inhibition [1]. Many medical agents such as antibacterials, antifungals, and anti-tumour drugs have been found to display hormetic response [2] with the earliest observations dating back to 1800s. In particular, low concentrations of certain antifungals were found to stimulate fungal growth [3] or metabolism [4] while inducing toxicity at high concentrations. From the early 1920s the concept of low-dose stimulation and high-dose toxicity of various chemical elements with respect to bacterial growth was widely recognised [5]. We now know that bacteria can exhibit hormetic response to a wide range of antibiotic drugs, regardless of their mode of action [6]. This phenomenon is also found in tumour cells exposed to anti-tumour drugs. In fact, hormesis has been observed in an astonishingly broad range of tumour types including pancreatic, colon and breast (reviewed in [7]).

Despite the overwhelming body of research, some dating back a century, that documents hormetic responses to a broad range of compounds, their clinical significance has only relatively recently come to the fore [2]. The consequence of hormesis could have a profound effect for human health [8, 9]. Drug concentration generally varies substantially within the human body and as drug gets cleared, the associated low concentration can in turn stimulate pathogen or tumour growth. Therefore understanding the mechanistic basis of hormesis is vital for both drug development and clinical practice.

The vast majority of targets for antibiotics, antifungals and anti-tumour drugs fall into the following categories: enzymes, receptors, transporters and DNA/RNA and the ribosome [10]. However how such drug-target interactions lead to hormesis remains poorly understood. The biological explanations put forward are overcompensation after a disruption of homeostasis (reviewed in [11]), direct stimulatory response [12], superimposition of different monotonic dose-response curves [13], or heterogenic susceptibility of different tissues to the same stimuli [14]. These explanations provide understanding of hormesis at a phenotypic level but lack understanding at the molecular level. Some inroads have also been made with respect to mammalian cells focusing on drug mechanisms mediated via receptor and/or cell signalling pathways (reviewed in [7]). For example, biphasic dose response could occur through interaction of two different receptor subtypes that mediate/activate opposing stimulatory and inhibitory pathways via the same antagonist [15]. However, hormetic response is a built in feature of such receptor mediated mechanisms rather than an emergent property of the underlying biological system.

An area of research where understanding of the mechanisms giving rise to hormesis is particularly lacking involves enzyme-targeting drugs. Known as enzyme inhibitors, they are designed to block enzyme activity leading to disruption of bacterial cell wall [16], fungal membranes [17] and fungal cell wall [18] as well as programmed tumour cell death [19], to name a few. With regards to hormetic dose-responses to antibiotics, a recent study focusing on inhibition of a specific enzyme, Dihydropteroate synthase, suggested the involvement of bacterial quorum sensing [20]. To our knowledge, mechanisms behind hormetic dose-response to enzyme-inhibiting antifungals are not known.

In recent years kinase inhibitors, a subset of enzyme inhibitors, have been shown to be very effective therapeutic agents in a broad range of diseases, including cancers. Amongst other enzyme inhibitors, significant attention has been focused on those inhibiting the mitogen-activated protein kinase (MAPK) pathway [21–24], which is of fundamental importance to human health as abnormal regulations of MAPK contribute to tumour progression [25].

The observations of hormesis in MAPKs as a result of inhibition of BRAF oncogene are widespread: low doses of RAF inhibitors designed to cease tumour proliferation [26] can cause a paradoxical activation of tumour cell activity through undesired MAPK up-regulation [8, 9, 27–32]. Current explanations of hormetic responses induced by RAF kinase inhibition involve complex phenomena affecting regulatory mechanisms, feedback pathways or enzymatic activity [33], making them difficult to generalise. More generally, enzyme competition for the same substrate was recently proposed as a simpler mechanism giving rise to hormetic effects of enzyme-targeting Alzheimer’s drugs [34].

In this paper we put forward a novel, simple but general mechanism driving hormetic responses in systems where an inhibitor acts on an enzyme. We develop a mathematical model based on a basic building block in intracellular signalling, namely a dual phosphorylation-dephosphorylation motif, to which a kinase inhibitor is applied. In a broader context, dual-phosphorylation can be found in diverse processes such as circadian rhythms [35], virulence regulation [36, 37], mitotic entry [38], transcription [39, 40], cytokine production [40], as well as in MAPK pathways which regulate primary cellular activities in eukaryotes including proliferation and programmed cell death [41, 42].

The model demonstrates that under certain conditions the steady state amount of the double-phosphorylated protein substrate in the cycle can substantially increase at low inhibitor doses compared to the base level without inhibition. Therefore the dose-response curve of the double-phosphorylated substrate possesses a hallmark of hormesis: it is upward sloping at low inhibitor doses and downward sloping at high inhibitor doses. The existence of hormesis in our model depends on the mechanism of inhibition and the dissociation rates of the kinase-substrate-inhibitor complexes. We also found that the magnitude of hormetic responses depends on the substrate-kinase ratio in a non-monotone way.

The benefits of our study are two-fold. Our mechanism is based on a principal component of intracellular signalling pathways, and as such has a potential broad applicability. Moreover having simple molecular understanding of the causes of hormetic responses can greatly improve the design of new drug compounds that avoid such responses.

## Materials and Methods

### The mathematical model

We consider a simple dual phosphorylation-dephosphorylation motif, whereby a distinct kinase protein is phosphorylating a separate protein substrate. Multiple phosphorylations can occur in close proximity or in diverse sites on a protein and here we focus on the former, instances of which can be found in activation of conventional MAPK enzymes [43], cell-cycle regulation via cyclin-dependent kinase 1 [44], regulation of other non-MAPK kinases [45] and ion channel trafficking [46]. The motif we consider is a subset of futile cycles [47, 48] also known as a single stage module in the context of MAPK pathways [49, 50]. Based on the experimental evidence for MAPK pathways [51–53] we assume that our motif follows a distributive mechanism consisting of two sequential phosphorylation steps and two sequential dephosphorylation steps that share the same intermediate mono-phosphorylated form. In particular, the protein substrate (C) is first converted into a mono-phosphorylated form (C_P_) and subsequently into a double-phosphorylated form (C_PP_), through a chain of reactions facilitated by a kinase (kin). Conversely C_PP_ is converted back to C_P_ which is subsequently converted to C, through a chain of reactions facilitated by a phosphatase (pho). In the distributive mechanism, the kinase(phosphatase) facilitates at most one phosphorylation (dephosphorylation) in each molecular encounter [48].

Therefore our dual phosphorylation-dephosphorylation motif can be described by the following reaction kinetic equations, which are a simplification of the reaction scheme described in [54]:

$$\begin{array}{lll} \text{C}+\text{kin} & \overset{k_{1}}{\underset{k_{-1}}{⇌}} & \text{C}\cdot \text{kin}\overset{k_{2}}{\to }{\text{C}}_{\text{P}}+\text{kin},\\ {\text{C}}_{\text{P}}+\text{kin} & \overset{k_{3}}{\underset{k_{-3}}{⇌}} & {\text{C}}_{\text{P}}\cdot \text{kin}\overset{k_{4}}{\to }{\text{C}}_{\text{PP}}+\text{kin},\\ {\text{C}}_{\text{PP}}+\text{pho} & \overset{k_{5}}{\underset{k_{-5}}{⇌}} & {\text{C}}_{\text{PP}}\cdot \text{pho}\overset{k_{6}}{\to }{\text{C}}_{\text{P}}+\text{pho},\\ {\text{C}}_{\text{P}}+\text{pho} & \overset{k_{7}}{\underset{k_{-7}}{⇌}} & {\text{C}}_{\text{P}}\cdot \text{pho}\overset{k_{8}}{\to }\text{C}+\text{pho}. \end{array}$$ (1)

Next we describe the assumptions behind the introduction of an inhibitor into Eq (1), based on the general modifier mechanism also known as hyperbolic or partial competitive inhibition [55]. We assume that the inhibitor (inh) is able to react with the kinase and the substrate-kinase intermediate complexes C · kin and C_P_ · kin according to the following inhibition scheme:

$$\begin{array}{lll} \text{kin}+\text{inh} & \overset{d_{f}}{\underset{d_{r}}{⇌}} & \text{kin}\cdot \text{inh},\\ \text{C}\cdot \text{kin}+\text{inh} & \overset{e_{1}}{\underset{e_{-1}}{⇌}} & \text{C}\cdot \text{kin}\cdot \text{inh}\overset{e_{2}}{\underset{e_{-2}}{⇌}}\text{C}+\text{kin}\cdot \text{inh},\\ {\text{C}}_{\text{P}}\cdot \text{kin}+\text{inh} & \overset{e_{3}}{\underset{e_{-3}}{⇌}} & {\text{C}}_{\text{P}}\cdot \text{kin}\cdot \text{inh}\overset{e_{4}}{\underset{e_{-4}}{⇌}}{\text{C}}_{\text{P}}+\text{kin}\cdot \text{inh}. \end{array}$$ (2)

The first- and second-order rates *k*_*i*_ and *e*_*i*_ in Eqs (1) and (2) and the association and dissociation rates *d*_*f*_ and *d*_*r*_ in Eq (2) are considered dimensionless. In our system intermediate substrate-kinase-inhibitor complexes are able to dissociate into a substrate and kinase-inhibitor complex with forward *e*_2_, *e*_4_ and backward *e*_−2_, *e*_−4_ rates [56].

The model describing the time evolution of the substrate, kinase, phosphatase and inhibitor concentrations is based on the law of mass action and assumes the total conservation of mass holds for all four compounds. The details of the system of 9 differential equations and the corresponding analysis are presented in S1 Appendix. (with Supplementary Tables A1 and A2 containing model parameter values). This model system is studied under steady state conditions, that is, when all concentrations of reactants have reached a dynamic equilibrium. Numerical simulations are conducted with Matcont, a continuation package in MATLAB used for numerical bifurcation analysis of ODEs [57].

## Results

In the absence of an inhibitor, the double phosphorylation motif Eq (1) can possess either a single or two stable steady states of the doubly-phosphorylated form of the substrate C_PP_, [50, 54, 58, 59]. Therefore in our study we consider two cases: first, when the motif Eq (1) is monostable and second, when this motif is bi-stable.

**Fig 1:**
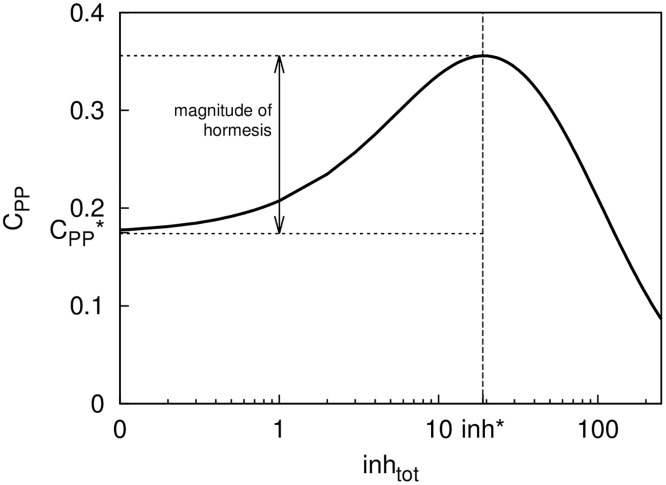
Dose response curve (log scale) for the double-phosphorylated substrate C_PP_ in the presence of an inhibitor (inh_tot_), in the case when the motif Eq (1) is monostable. In the absence of the inhibitor the stable steady state value of the double-phosphorylated substrate is denoted by C_PP_*. With the inhibitor present, the dose response exhibits hormetic properties whereby for sufficiently low inhibitor doses (inh_tot_<inh*) the computed steady state values of C_PP_ increase monotonically, before monotonically decreasing for inhibitor concentrations inh_tot_>inh*. The magnitude of hormetic response is calculated as a difference between the C_PP_ value at inh* and C_PP_*.

In the case of a single stable steady state (C_PP_*) in the absence of an inhibitor, we find that C_PP_ can exhibit biphasic (or hormetic) response to an inhibitor as illustrated in Fig 1. In particular, the observed dose-response curve in the presence of an inhibitor has an inverted U-shape: for sufficiently low inhibitor doses the computed steady-state values of C_PP_ increase monotonically, while for sufficiently large inhibitor doses, the computed steady-state values of C_PP_ monotonically decrease.

Moreover, by making simplifying assumptions that *e*_−2_ = *e*_−4_ = 0, *e*_2_ >>*e*_1_, *e*_4_ >>*e*_3_ and the inhibitor has fast off rate, we can analytically derive the slope of the dose-response curve, in other words the slope of the relationship between the steady-state value of C_PP_ and the total amount of inhibitor at low doses (see S1 Appendix for details). This allows us to identify two primary factors necessary for the hormesis to be observed:

(C1) the strong dissociation effect of intermediate substrate-kinase-inhibitor complexes C · kin · inh and C_P_ · kin · inh, corresponding to *e*_2_, *e*_4_ > 0,
(C2) large dissociation rate of kinase-inhibitor complexes.

Note that the hormesis is still observed in numerical simulations when *e*_−2_, *e*_−4_ > 0 (Fig A1 in S1 Appendix).

**Fig 2:**
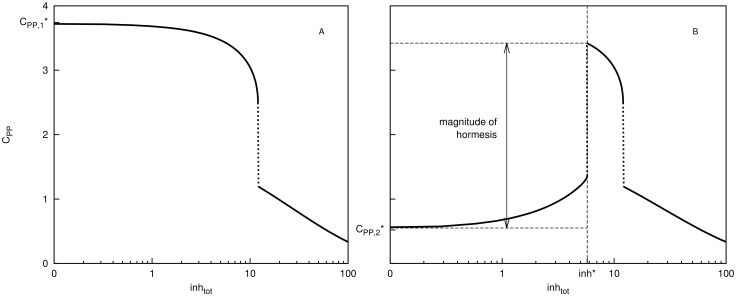
Dose response curve (log scale) for the double-phosphorylated substrate C_PP_ in the presence of an inhibitor (inh_tot_), in the case when the motif Eq (1) is bistable. In the absence of the inhibitor there are two stable steady states of the double-phosphorylated substrate (A) C_PP,1_* and (B) C_PP,2_*. Cell populations at these two steady state will react differently to the presence of an inhibitor: (A) cells at C_PP,1_* will exhibit a monotone dose-response while (B) cells at C_PP,2_* will exhibit a hormetic dose response whereby for sufficiently low inhibitor doses (inh_tot_<inh*) the computed steady state values of C_PP_ increase monotonically, before monotonically decreasing for inhibitor concentrations inh_tot_>inh*. The dotted lines indicate a discontinuous jump in the steady state values of C_PP_ in the presence of the inhibitor. The magnitude of hormetic response is calculated as a difference between the C_PP_ value at inh* and C_PP,2_*.

In addition the above conditions (C1-C2) can also be used to forecast the presence of a hormetic dose response in the second case under our consideration, namely when in the absence of an inhibitor the motif Eq (1) has two stable steady states C_PP,1_* (Fig 2A) and C_PP,2_* (Fig 2B). In this case the numerical simulations predict that cells with high base level of double-phosphorylated substrate will respond differently to inhibition from the cells with low base level of double-phosphorylated substrate. In particular, cells with initially high levels of C_PP_ (at steady state C_PP,1_*) will exhibit a monotone decreasing dose-response (Fig 2A) while cells with low initial levels of C_PP_ (at steady state C_PP,2_*) will exhibit a hormetic response (Fig 2B).

The magnitude of hormetic response can differ between the mono- and bi-stable cases under consideration as illustrated in Figs 1 and 2B. In the mono-stable case the C_PP_ value at dose inh* is approximately two-fold higher compared to the base level C_PP_* value in the absence of an inhibitor (Fig 1). In the bi-stable case the C_PP_ value at dose inh* is approximately six-fold higher than the base level C_PP,2_* value in the absence of an inhibitor (Fig 2B).

**Fig 3:**
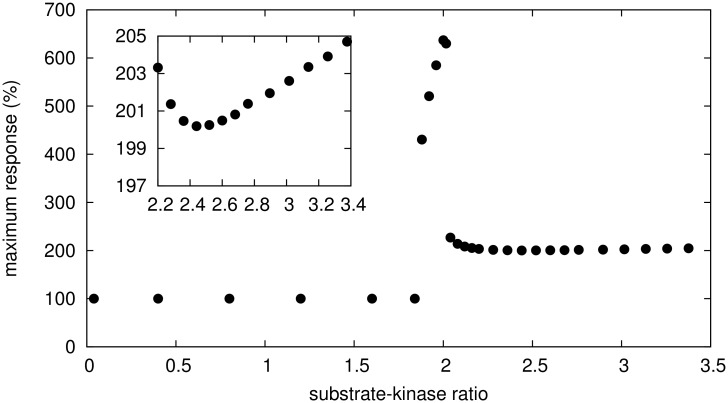
Maximum hormetic response. The maximum hormetic response is calculated as the maximal increase in C_PP_ over all inhibitor doses, relative to the base line amount of C_PP_ in the absence of inhibition. Since the baseline is represented at a level of 100%, hormesis is present if the maximum response strictly exceeds the baseline (> 100%). This maximum response is plotted (black dots) as a function of different substrate-kinase ratios achieved by varying the total mass of substrate and keeping the total mass of kinase constant.

In general, we find that the ratio of total mass of protein substrate to kinase mass influences the magnitude of hormetic response in a non-monotone way as shown in Fig 3. For sufficiently small substrate-kinase ratio, a hormetic response is not observed (absence of hormesis is labelled as 100% response in Fig 3 because the maximal response is equal to the baseline of no inhibition). However, the hormetic response increases sharply as the substrate-kinase ratio increases. Further increases of this ratio lead to a sharp decline in the magnitude of hormetic response, which continues to increase slowly for sufficiently large substrate-kinase ratios (see Fig 3 inset). Therefore, the magnitude of hormetic response peaks at intermediate values of the substrate-kinase ratio, as frequently observed in the MAPK pathway [60] for example, while hormesis is not observed for low substrate-kinase ratios.

## Discussion

Hormetic responses to enzyme-targeting drugs have been observed in both prokaryotes [20, 61, 62] and eukaryotes [8, 9, 27, 31, 32] but the mechanistic understanding behind such responses is still lacking. In this paper we focus on eukaryotic cells and propose a novel, simple but general mechanism that could give rise to hormesis in systems where an inhibitor acts on an enzyme.

At the core of our newly-proposed mechanism is one of the basic building blocks in intracellular signalling, the dual phosphorylation-dephosphorylation motif, found in diverse regulatory processes including MAPK pathways which control cell proliferation and programmed cell death in eukaryotes [41, 42]. We analytically derive conditions that lead to hormetic dose-response of the doubly-phosphorylated substrate in the presence of a kinase inhibitor. The conditions required for hormesis to be observed are surprisingly simple and involve two main factors: (C1) strong dissociation effect of intermediate substrate-kinase-inhibitor complexes and (C2) large dissociation rate of kinase-inhibitor complexes.

Crystallographic studies of kinase inhibitors bound to their targets demonstrate that a number of different conformational states can be induced. Type 1 kinase inhibitors are defined as binding the kinase in its active conformation and crystal structures of ternary complexes of ATP analogues bound with substrate peptides are reported (for review see [63, 64]). Indeed it is not uncommon for crystal systems of substrate peptide complexes to be used in Structure Based Design campaigns to develop Type 1 kinase inhibitors [65].

**Fig 4:**
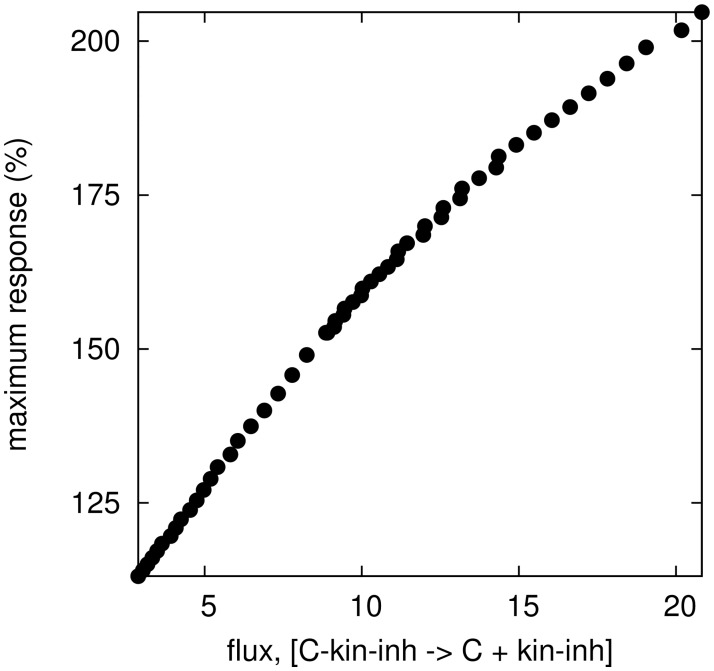
Relationship between the maximum hormetic response and the flux C · kin · inh → C + inh · kin. The maximum hormetic response is calculated as the maximal increase in C_PP_ over all inhibitor doses, relative to the base line amount of C_PP_ in the absence of inhibition. Since the baseline is represented at a level of 100%, hormesis is present if the maximum response strictly exceeds the baseline (> 100%). The flux is computed as *e*_2_[C · kin · inh] − *e*_−2_[C][kin · inh] using the steady state values [C](inh*), [kin · inh](inh*), [C · kin · inh](inh*) with inh* being the dose with the maximum hormetic response (see Fig 1). As *e*_−2_ increases, the flux decreases to 0.

Given the fundamental nature of the dual phosphorylation-dephosphorylation motif and the relative simplicity of the derived conditions necessary to observe hormesis, why was this mechanism previously overlooked in theoretical literature? A further examination of the (C1) condition could provide a potential answer. In general, when considering partial competitive enzyme inhibition [55] as we do here, classical enzyme kinetics literature [55, 56] assumes not only equilibrium concentrations of different enzyme species but it also assumes that at those equilibrium concentrations there is no flux through substrate-kinase-inhibitor complexes. However, we find that in our study as flux decreases the maximum hormetic response also decreases (Fig 4) indicating that under the no-flux assumption, hormetic responses could be overlooked.

Once a new mechanism is proposed to explain a particular biological phenomenon, ideally it should be put to test. However, there are a number of difficulties associated with *in vitro* tests of our model predictions. First, biochemical assays involved with *in vitro* studies are not standardised and vary between research groups, making comparisons between already published observations difficult. Second, testing our model predictions requires measurements of single and double phosphorylation outputs, this could be problematic as antibody specificity required to distinguish these outputs might not readily be available. This would particularly be relevant for systems where phosphorylation sites are situated close together. Third, ensuring that the condition for observing hormesis *e*_2_, *e*_4_ > 0 is satisfied experimentally is challenging as kinase biochemical assays would not usually include phosphatase activity. Furthermore varying rates of reactions individually or measuring fluxes in such systems is equally difficult.

Having discussed difficulties associated with testing our model in reductionist *in vitro* systems, we next consider whether these difficulties could be overcome with a cell-based experimental systems. In particular, our model predicts that hormetic dose-response could be a wide-spread feature of MAPK pathways when exposed to enzyme inhibitors. However we argue here that the non-trivial biphasic dose-response associated with hormesis might often be overlooked when performing experiments at cell population level, as we now discuss.

Consider the case where in the absence of an inhibitor, the double phosphorylation motif Eq (1) possesses two stable steady states of the doubly-phosphorylated form of the substrate C_PP_. This means that tumour cells within a population can be grouped into two types: type-1 cells with ‘high’ C_PP_ and type-2 cells with ‘low’ C_PP_. In reality these heterogeneous cell phenotypes can emerge not only due to multistability of the system [50, 54] but also due to stochastic fluctuations which lead to different concentrations of the the total protein substrate [66, 67]. In general, an untreated tumour is likely to harbour different proportions of cells in different phenotypic states [68].

We show that different cell types can respond differently to the presence of an inhibitor. Namely, our model predicts that in certain cases cells with initially high levels of C_PP_ (at steady state C_PP,1_*) will exhibit a monotone decreasing dose-response (Fig 2A) while cells with low initial levels of C_PP_ (at steady state C_PP,2_*) will exhibit a hormetic response (Fig 2B). This has an important consequence for measuring C_PP_ at a population level as it is frequently done [69], as well as determining inhibitory concentrations (IC). Such consequences are best illustrated with the following example.

**Fig 5:**
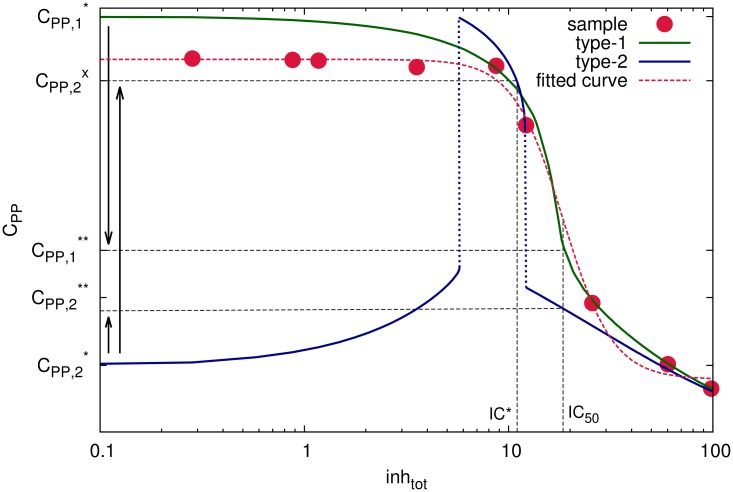
Heterogeneous populations. Dose response curves of type-1 (green line) and type-2 (blue line) cells in the presence of an inhibitor (inh_tot_), in the case when motif Eq (1) is bistable. A logistic curve (red line) is fitted to sample points (red dots) generated numerically from a population containing 88% type-1 cells and 12% type-2 cells. The logistic fit to data is used to estimate IC_50_ of the total population (see Supplementary Information). In the absence of the inhibitor type-1 cells are at C_PP,1_* stead state while type-2 cells are at C_PP,2_*. In the presence of the inhibitor at IC_50_, while the total population exhibits 50% inhibition, the same concentration has the opposing effects on the two sub-populations. In particular, type-1 cells are inhibited, which can be deduced from the observation that the steady state C_PP,1_** calculated at the population-level IC_50_ is lower than the steady state C_PP,1_* calculated in the absence of the inhibitor. Contrary to this type-2 cells are stimulated since the steady state C_PP,2_** calculated at the population-level IC_50_ is higher than the steady state C_PP,2_* calculated in the absence of the inhibitor. This stimulatory effect is amplified even further for IC*<IC_50_, as seen by comparing the relatively high values of the steady state C_PP,2_^x^ at IC* to the relatively low values of the steady state C_PP,2_* in the absence of the inhibitor.

Let us assume, for example, that 88% of the tumour cells are type-1 cells and 12% of the tumour cells are type-2 cells. We can then simulate our model to generate dose response curves of C_PP_ for both type-1 (Fig 5, green line) and type-2 (Fig 5, blue line) phenotypes. In addition, we can also numerically generate sampled values of the combined dose response of the entire population as would be measured, for instance, in a western blot or population-based imaging assay for C _PP_ (Fig 5, red dots). By fitting a logistic curve to the sampled values of the combined dose response (Fig 5, red dashed line) we can estimate the inhibitor concentration causing 50% inhibition of the entire population, denoted IC_50_. However, the same inhibitor concentration has the opposing effects on the two sub-populations: while it inhibits type-1 cells, it actually stimulates type-2 cells. This can be observed by comparing steady-state values of C_PP_ in the absence of inhibition (C_PP,1_* for type-1 and C_PP,2_* for type 2) to the steady-state values of C_PP_ in the presence of the inhibitor (C_PP,1_** for type-1 and C_PP,2_** for type 2) at the IC_50_ concentration estimated for the entire population (Fig 5). In particular, the inhibition of type-1 cells can be seen from C_PP,1_*>C_PP,1_** while the stimulation of type-2 cells can be seen from C_PP,2_*<C_PP,2_**. Such unexpected stimulatory effects of the population-level IC_50_ exerted on type-2 sub-population could be further amplified when taking into account the imperfect drug penetration in a tumour [70]. In that case tumour cells would actually experience a lower inhibitor concentration IC*<IC_50_, which could lead to significant increases in steady-state values of C_PP_ (denoted by C_PP,2_^x^ in Fig 5), compared to the steady-state values of C_PP_ in the absence of inhibition (denoted by C_PP,2_* in Fig 5). A numerical example with balanced type-1 and type-2 cell populations is presented in Fig A5 of S1 Appendix, showing that in this case it is also possible to mask the hormetic response at the population level, although the maximal hormetic response of the type-2 cells at the corresponding IC_50_ is substantially lower.

The presence of hormetic responses to an inhibitor which are masked at a population level could, therefore, complicate the interpretation of, and understanding gained from, preclinical models. Such complex sub-population effects have been noted for example in the NF-κB pathway, controlling DNA transcription, cytokine production and cell survival [71]. In particular, studies have shown that observing non-synchronous cells at a population level may under-represent oscillatory behaviour of nuclear shuttling [40, 72–74].

Examining hormetic responses at single-cell level could substantially improve detection rates as well as help identify mechanisms driving hormesis. However, while measuring and analysing single-cell bacterial dose response to antibiotics is already feasible [75], such methodology has rarely been implemented for studying dose-responses of tumour cells. Therefore, a wider application of single-cell dose-response techniques used for prokaryotes to tumour cells will greatly enhance our understanding of hormesis in cancer settings.

The conclusions of our study are based on the assumption that the dual phosphorylation-dephosphorylation motif presented in Eq (1) follows a distributive mechanism, whereby kinase (phosphatase) facilitates at most one phosphorylation (dephosphorylation) in each molecular encounter. This is motivated by the experimental evidence for MAPK pathways [51–53]. However, phosphorylation and dephosphorylation cycles can also follow a processive mechanism in which the kinase (phosphatase) facilitates two or more phosphorylations (dephosphorylations) before the final product is released [48]. In addition, a quasi-processive mechanism has been recently proposed to operate under the physiological condition of molecular crowding, which is a critical factor converting distributive into processive phosphorylation [76–78]. Our model can readily be extended to consider these alternative scenarios.

The findings presented here are relevant to applications in drug discovery relating to MAPK inhibition. Whereas inhibitors are specifically designed to target and suppress various stages in the MAPK pathways, the hormesis phenomenon leads to the opposite effect lowering the effectiveness of the compound and potentially leading to failure in the clinic [8, 9, 32]. Therefore, understanding mechanisms that lead to this undesired effect is important for designing inhibitors that would avoid them. Indeed, a recent study proposed a novel inhibitor, designed specifically to avoid MAPK activation at low-doses [79].

Our study could help achieve a similar goal. In particular, a straight forward approach to mitigate the risk of hormetic response is to favour inhibitor mechanisms of action for which this is impossible under our model. Protein substrate competitive inhibitors is one such example as these would generally, through steric hindrance, prohibit the formation of the necessary tertiary complex. In practice, structural biology can be employed to confirm that substrate and inhibitor complexes are mutually exclusive.

Overall, we argue that mathematical models are particularly useful tools in the drug-discovery process. Given the difficulties associated with measuring hormetic responses empirically be it with reductionist *in vitro* biochemical assays or cell based systems, the involvement of mathematical models in this process is of paramount importance. What we demonstrate here is that theoretical models classically make assumptions that immediately discount the possibility of observing hormetic responses in cell signalling pathways in the presence of inhibitors. Namely the assumption of no flux through substrate-kinase-inhibitor complex in motif Eq (2) is widespread in theoretical literature despite the lack of empirical support. It is, therefore, crucial that model assumptions are regularly challenged so that important behaviours are not overlooked.

## Supporting Information

S1 Appendix The file contains a detailed mathematical model describing the time evolution of the substrate, kinase, phosphate and inhibitor concentrations, alongside the corresponding analysis and model parametrisation. (PDF)
