# Supplementary material for: Kinase Inhibition Leads to Hormesis in a Dual Phosphorylation-Dephosphorylation Cycle
Source: PLoS Comput Biol. 2016 Nov 29;12(11):e1005216. doi: 10.1371/journal.pcbi.1005216 (PMC5127489; doi:10.1371/journal.pcbi.1005216)
Supplement: S1 Appendix — (PDF) [file pcbi.1005216.s001.pdf]

SUPPLEMENTARY INFORMATION: KINASE INHIBITION LEADS TO HORMESIS IN A DUAL PHOSPHORYLATION-DEPHOSPHORYLATION CYCLE

P. RASHKOV, I.P. BARRETT, R.E. BEARDMORE, C. BENDTSEN, I. GUDELJ

1. Mathematical model

The kinetics of the reaction scheme for the dual phosphorylation-dephosphorylation cycle subject to kinase inhibition (presented in (1-2) of the Main Text) are used to develop a model of temporal dynamics for 13 variables representing concentrations of the following compounds: substrate [C], and mono- and double-phosphorylated substrate [$\mathrm{C_P}$], [$\mathrm{C_{PP}}$], kinase [kin], phosphatase [pho] and inhibitor [inh] in free form, and substrate-kinase complexes [C·kin], [$\mathrm{C_P}$·kin], substrate-phosphatase complexes [$\mathrm{C_P}$·pho], [$\mathrm{C_{PP}}$·pho], kinase-inhibitor complex [kin·inh] and substrate-kinase-inhibitor complexes [C·kin·inh], [$\mathrm{C_P}$·kin·inh]. These concentrations are subject to the mass conservation conditions for substrate, kinase, phosphatase and inhibitor of the form

$$
\begin{aligned}
(1)\qquad S_{tot} &= [\mathrm{C}] + [\mathrm{C_P}] + [\mathrm{C_{PP}}] + [\mathrm{C}\cdot\mathrm{kin}] + [\mathrm{C}\cdot\mathrm{kin}\cdot\mathrm{inh}] \\
&\quad + [\mathrm{C_P}\cdot\mathrm{kin}] + [\mathrm{C_P}\cdot\mathrm{kin}\cdot\mathrm{inh}] \\
&\quad + [\mathrm{C_P}\cdot\mathrm{pho}] + [\mathrm{C_{PP}}\cdot\mathrm{pho}], \\
(2)\qquad K_{tot} &= [\mathrm{kin}] + [\mathrm{kin}\cdot\mathrm{inh}] + [\mathrm{C}\cdot\mathrm{kin}] + [\mathrm{C}\cdot\mathrm{kin}\cdot\mathrm{inh}] \\
&\quad + [\mathrm{C_P}\cdot\mathrm{kin}] + [\mathrm{C_P}\cdot\mathrm{kin}\cdot\mathrm{inh}], \\
(3)\qquad P_{tot} &= [\mathrm{pho}] + [\mathrm{C_P}\cdot\mathrm{pho}] + [\mathrm{C_{PP}}\cdot\mathrm{pho}], \\
(4)\qquad \mathrm{inh_{tot}} &= [\mathrm{inh}] + [\mathrm{C}\cdot\mathrm{kin}\cdot\mathrm{inh}] + [\mathrm{C_P}\cdot\mathrm{kin}\cdot\mathrm{inh}] + [\mathrm{kin}\cdot\mathrm{inh}],
\end{aligned}
$$

where the total masses $P_{tot}, K_{tot}, S_{tot}, \mathrm{inh_{tot}}$ are non-negative.

The temporal dynamics of the concentrations using the law of mass action is represented in the following system of nine ordinary differential equations (ODEs)

$$
(5)\qquad
\begin{aligned}
[\mathrm{C_P}]' &= k_2[\mathrm{C}\cdot\mathrm{kin}] - k_3[\mathrm{C_P}][\mathrm{kin}] + k_{-7}[\mathrm{C_P}\cdot\mathrm{pho}] - k_7[\mathrm{C_P}][\mathrm{pho}] \\
&\quad + k_{-3}[\mathrm{C_P}\cdot\mathrm{kin}] + k_6[\mathrm{C_{PP}}\cdot\mathrm{pho}] + e_4[\mathrm{C_P}\cdot\mathrm{kin}\cdot\mathrm{inh}] \\
&\quad - e_{-4}[\mathrm{C_P}][\mathrm{kin}\cdot\mathrm{inh}], \\
[\mathrm{C_{PP}}]' &= k_4[\mathrm{C_P}\cdot\mathrm{kin}] + k_{-5}[\mathrm{C_{PP}}\cdot\mathrm{pho}] - k_5[\mathrm{C_{PP}}][\mathrm{pho}], \\
[\mathrm{C}\cdot\mathrm{kin}]' &= k_1[\mathrm{C}][\mathrm{kin}] + e_{-1}[\mathrm{C}\cdot\mathrm{kin}\cdot\mathrm{inh}] - (k_{-1} + k_2 + e_1[\mathrm{inh}])[\mathrm{C}\cdot\mathrm{kin}], \\
[\mathrm{C_P}\cdot\mathrm{kin}]' &= k_3[\mathrm{C_P}][\mathrm{kin}] + e_{-3}[\mathrm{C_P}\cdot\mathrm{kin}\cdot\mathrm{inh}] - (k_{-3} + k_4 + e_3[\mathrm{inh}])[\mathrm{C_P}\cdot\mathrm{kin}], \\
[\mathrm{kin}\cdot\mathrm{inh}]' &= d_f[\mathrm{kin}][\mathrm{inh}] + e_2[\mathrm{C}\cdot\mathrm{kin}\cdot\mathrm{inh}] + e_4[\mathrm{C_P}\cdot\mathrm{kin}\cdot\mathrm{inh}] - d_r[\mathrm{kin}\cdot\mathrm{inh}] \\
&\quad - e_{-2}[\mathrm{C}][\mathrm{kin}\cdot\mathrm{inh}] - e_{-4}[\mathrm{C_P}][\mathrm{kin}\cdot\mathrm{inh}], \\
[\mathrm{C}\cdot\mathrm{kin}\cdot\mathrm{inh}]' &= e_1[\mathrm{C}\cdot\mathrm{kin}][\mathrm{inh}] + e_{-2}[\mathrm{C}][\mathrm{kin}\cdot\mathrm{inh}] - (e_{-1} + e_2)[\mathrm{C}\cdot\mathrm{kin}\cdot\mathrm{inh}], \\
[\mathrm{C_P}\cdot\mathrm{kin}\cdot\mathrm{inh}]' &= e_3[\mathrm{C_P}\cdot\mathrm{kin}][\mathrm{inh}] + e_{-4}[\mathrm{C_P}][\mathrm{kin}\cdot\mathrm{inh}] - (e_{-3} + e_4)[\mathrm{C_P}\cdot\mathrm{kin}\cdot\mathrm{inh}], \\
[\mathrm{C_{PP}}\cdot\mathrm{pho}]' &= k_5[\mathrm{C_{PP}}][\mathrm{pho}] - (k_{-5} + k_6)[\mathrm{C_{PP}}\cdot\mathrm{pho}], \\
[\mathrm{C_P}\cdot\mathrm{pho}]' &= k_7[\mathrm{C_P}][\mathrm{pho}] - (k_{-7} + k_8)[\mathrm{C_P}\cdot\mathrm{pho}].
\end{aligned}
$$

In this system $[\cdot]$ denotes the concentration of each compound and $'$ represents the time derivative. The reaction rates k_i, e_i, d_f, d_r are non-negative.

Due to the constraints (1)–(4), system (5) does not include the equations for the temporal behaviour of [C], [kin], [pho] and [inh] as these are determined by the mass conservation laws, in fact, there are 9 equations because there are 9 degrees of freedom (= 13 variables – 4 constraints).

2. Analytical study

Of interest are the steady states of the system (5) as functions of the total inhibitor dose inh_{tot}, and especially that of the doubly-phosphorylated substrate, C_{PP}. In particular, our aim is to trace the dose response curve of the motif by plotting the steady state values of C_{PP} versus inh_{tot}. Monitoring the amount of the doubly-phosphorylated substrate is of importance in the MAPK pathway as C_{PP} is required for the pathway activation [4, 8, 9].

Since system (5) is a high-dimensional nonlinear system, a direct analytical approach is not feasible and several assumptions will be made in order to analyse its behaviour. However, as numerical computations show (see Fig. A1), the full system has similar characteristics as the simplified system.

Steady state equations. Denote $x_1 = [\text{C}], x_2 = [\text{C}_\text{P}], x_3 = [\text{C}_{\text{PP}}], x_4 = [\text{C}\cdot\text{kin}], x_5 = [\text{C}_\text{P}\cdot\text{kin}], y_1 = [\text{kin}\cdot\text{inh}], y_2 = [\text{C}\cdot\text{kin}\cdot\text{inh}], y_3 = [\text{C}_\text{P}\cdot\text{kin}\cdot\text{inh}],\ z_1 = [\text{C}_\text{P}\cdot\text{pho}], z_2 = [\text{C}_{\text{PP}}\cdot\text{pho}]$.

Setting all time derivatives in (5) to zero produces the following nonlinear system

$$0 = k_2x_4 - k_3x_2[\text{kin}] + k_{-7}z_1 - k_7x_2[\text{pho}] + k_{-3}x_5 + k_6z_2 + e_4y_3 - e_{-4}x_2y_1, \tag{6a}$$

$$0 = k_4x_5 + k_{-5}z_2 - k_5x_3[\text{pho}], \tag{6b}$$

$$0 = k_1x_1[\text{kin}] + e_{-1}y_2 - (k_{-1} + k_2 + e_1[\text{inh}])x_4, \tag{6c}$$

$$0 = k_3x_2[\text{kin}] + e_{-3}y_3 - (k_{-3} + k_4 + e_3[\text{inh}])x_5, \tag{6d}$$

$$0 = d_f[\text{kin}][\text{inh}] + e_2y_2 + e_4y_3 - d_ry_1 - e_{-2}x_1y_1 - e_{-4}x_2y_1, \tag{6e}$$

$$0 = e_1x_4[\text{inh}] + e_{-2}x_1y_1 - (e_{-1} + e_2)y_2, \tag{6f}$$

$$0 = e_3x_5[\text{inh}] + e_{-4}x_2y_1 - (e_{-3} + e_4)y_3, \tag{6g}$$

$$0 = k_5x_3[\text{pho}] - (k_{-5} + k_6)z_2, \tag{6h}$$

$$0 = k_7x_2[\text{pho}] - (k_{-7} + k_8)z_1, \tag{6i}$$

whose solutions are the steady states of the original system (5). Now we let

$$\alpha_1 = \frac{e_1}{e_{-1} + e_2}, \quad \alpha_2 = \frac{e_3}{e_{-3} + e_4}, \quad \beta_3 = \frac{k_5}{k_{-5} + k_6}, \quad \beta_4 = \frac{k_7}{k_{-7} + k_8},$$

and focus on the behaviour of steady-state value x_3 of C_{PP} as the inhibitor dose inh_{tot} increases from 0.

Assumptions. In the analysis we make the following assumptions in order to analyse the solutions of system (6):

(a1) $e_{-2}, e_{-4} = 0$,
(a2) $e_2 >> e_1$ and $e_4 >> e_3$,
(a3) $d_r/d_f >> 1$.

Next step is to compute the steady state values of the triple substrate-kinase-inhibitor complexes.

Inhibitor complexes. Observe that assumption (a2)–(a3) effectively mean that the condition of total mass conservation for the inhibitor (4) is relaxed when $\mathrm{inh_{tot}} \approx 0$. In fact, solving for y_2, y_3 from (6f), (6g) gives

$$y_2 = \alpha_1 x_4[\mathrm{inh}], \quad y_3 = \alpha_2 x_5[\mathrm{inh}]. \tag{7}$$

As assumption (a2) implies α_1, α_2 are small, the steady state values of y_2, y_3 (that is, the inhibitor complexes $[\mathrm{C}\cdot\mathrm{kin}\cdot\mathrm{inh}], [\mathrm{C_P}\cdot\mathrm{kin}\cdot\mathrm{inh}]$) are also small compared to [inh].

Substituting (7) sequentially into (6f), (6g), (6e) gives the following relationships:

$$x_4 = \frac{k_1 x_1[\mathrm{kin}]}{k_{-1} + k_2 + e_2\alpha_1[\mathrm{inh}]},$$
$$x_5 = \frac{k_3 x_2[\mathrm{kin}]}{k_{-3} + k_4 + e_4\alpha_2[\mathrm{inh}]},$$
$$y_1 = \frac{[\mathrm{kin}]}{d_r}\left(d_f[\mathrm{inh}] + \frac{\alpha_1 k_1 x_1[\mathrm{inh}]}{k_{-1} + k_2 + e_2\alpha_1[\mathrm{inh}]} + \frac{\alpha_2 k_3 x_2[\mathrm{inh}]}{k_{-3} + k_4 + e_4\alpha_2[\mathrm{inh}]}\right).$$

Assumptions (a2)-(a3) also imply that the steady state concentration y_1 of $[\mathrm{kin}\cdot\mathrm{inh}]$ is small. Thus, the condition (4) simplifies to $[\mathrm{inh}] \approx \mathrm{inh_{tot}}$ and therefore $[inh] := \mathrm{inh_{tot}}$ will be used for the rest of this argument.

Phosphatase complexes. Next, we solve for the equilibrium values of unbound phosphatase [pho] along the framework of Michaelis-Menten kinetics. The steady state concentration of the phosphatase complexes z_1, z_2 are derived from (6h), (6i),

$$z_1 = \beta_4 x_2[\mathrm{pho}], \quad z_2 = \beta_3 x_3[\mathrm{pho}],$$

and using the total mass conservation relation for the phosphatase (3)

$$[\mathrm{pho}] + z_1 + z_2 = P_{tot},$$

one can solve for [pho],

$$[\mathrm{pho}] = \frac{P_{tot}}{1 + \beta_3 x_3 + \beta_4 x_2}.$$

Kinase complexes. Using the total mass conservation relation for the kinase (2)

$$x_4 + x_5 + y_1 + y_2 + y_3 + [\mathrm{kin}] = K_{tot},$$

we solve for the amount unbound kinase [kin]

$$[\mathrm{kin}] = \frac{K_{tot}}{1 + \frac{d_f}{d_r}\mathrm{inh_{tot}} + \frac{x_1}{\rho_1(\mathrm{inh_{tot}})} + \frac{x_2}{\rho_2(\mathrm{inh_{tot}})}},$$

where $\rho_{1,2}$ are macroscopic rate constants, functions of $\mathrm{inh_{tot}}$

$$\rho_1(\mathrm{inh_{tot}}) = \frac{k_{-1} + k_2 + \alpha_1 e_2 \mathrm{inh_{tot}}}{k_1(1 + \frac{\alpha_1 e_2}{d_r}\mathrm{inh_{tot}} + \alpha_1\mathrm{inh_{tot}})},$$
$$\rho_2(\mathrm{inh_{tot}}) = \frac{k_{-3} + k_4 + \alpha_2 e_4 \mathrm{inh_{tot}}}{k_3(1 + \frac{\alpha_2 e_4}{d_r}\mathrm{inh_{tot}} + \alpha_2\mathrm{inh_{tot}})}.$$

Solving for the steady-state value of C_{PP}. To find the value of x_3 we substitute the newly-determined expressions for x_5, z_2 and β_3 into (6b) to obtain

$$0 = \frac{k_3 k_4 x_2[\text{kin}]}{k_{-3} + k_4 + e_4\alpha_2 \text{inh}_{\text{tot}}} + (\frac{k_{-5}k_5}{k_{-5}+k_6} - k_5)x_3[\text{pho}],$$

which is equivalent to

$$0 = \frac{k^{cat}(\text{inh}_{\text{tot}})K_{tot}\frac{x_2}{\rho_2(\text{inh}_{\text{tot}})}}{1 + \frac{\text{inh}_{\text{tot}}d_f}{d_r} + \frac{x_1}{\rho_1(\text{inh}_{\text{tot}})} + \frac{x_2}{\rho_2(\text{inh}_{\text{tot}})}} - \frac{k_6\beta_3 P_{tot}x_3}{1+\beta_3 x_3 + \beta_4 x_2}, \tag{8}$$

with a catalytic constant dependent on inh_{tot},

$$k^{cat}(\text{inh}_{\text{tot}}) = \frac{k_4}{1 + \frac{\alpha_2 e_4}{d_r}\text{inh}_{\text{tot}} + \alpha_2 \text{inh}_{\text{tot}}}.$$

Equation (8) presents a relationship between the estimated steady state value of x_3 and inh_{tot} which is analysed for small $\text{inh}_{\text{tot}} > 0$. Observe that the latter summand in equation (8) is monotone in x_3 and for small β_4 (compare the rates for dephosphorylation of the mono-phosphorylated substrate in [8, 9]), the effect of x_2 on this term will be negligible.

Now, in equation (8) consider the former summand's dependence on inh_{tot} under the assumption that x_1, x_2 are slowly-varying in inh_{tot}. First, we set

$$M(\text{inh}_{\text{tot}}) = \frac{k^{cat}(\text{inh}_{\text{tot}})\frac{x_2}{\rho_2(\text{inh}_{\text{tot}})}}{1 + \frac{d_f \text{inh}_{\text{tot}}}{d_r} + \frac{x_1}{\rho_1(\text{inh}_{\text{tot}})} + \frac{x_2}{\rho_2(\text{inh}_{\text{tot}})}}.$$

Then the partial derivative M' in inh_{tot} at the steady state $(\text{inh}_{\text{tot},0}; \hat{x}_1, \hat{x}_2)$ is computed as

$$M'(\text{inh}_{\text{tot},0}) = x_2 \frac{\nu_1(\text{inh}_{\text{tot},0})}{\nu_2(\text{inh}_{\text{tot},0})},$$

with

$$\begin{aligned} \nu_1(h) &= \left(\frac{k^{cat}}{\rho_2}\right)' \left(1 + \frac{d_f h}{d_r} + \frac{\hat{x}_1}{\rho_1(h)} + \frac{\hat{x}_2}{\rho_2(h)}\right) \\ &\quad - \frac{k^{cat}}{\rho_2}\left(\frac{d_f}{d_r} - \frac{\hat{x}_1\rho_1'(h)}{\rho_1(h)^2} - \frac{\hat{x}_2\rho_2'(h)}{\rho_2(h)^2}\right), \\ \nu_2(h) &= \left(1 + \frac{d_f h}{d_r} + \frac{\hat{x}_1}{\rho_1(h)} + \frac{\hat{x}_2}{\rho_2(h)}\right)^2. \end{aligned}$$

Since the denominator $\nu_2 > 0$, the sign of the partial derivative M' depends on the sign of the expression $\nu_1(\text{inh}_{\text{tot},0})$. Hence, it suffices to examine the sign of

$$\begin{aligned} \nu_1(h) = &-\frac{k_3 k_4 \alpha_2 e_4}{(k_{-3} + k_4 + \alpha_2 e_4 h)^2}\left(1 + \frac{d_f h}{d_r} + \frac{\hat{x}_1}{\rho_1} + \frac{\hat{x}_2}{\rho_2}\right) - \frac{k^{cat}}{\rho_2}\frac{d_f}{d_r} \\ &+ \frac{k^{cat}}{\rho_2}\frac{\hat{x}_1\rho_1'}{\rho_1^2} + \frac{k^{cat}}{\rho_2}\frac{\hat{x}_2\rho_2'}{\rho_2^2}. \end{aligned} \tag{9}$$

In (9) the argument h of $\rho_{1,2}$ is omitted for the sake of clarity of presentation. We note that the first two summands on the right-hand side in (9) are unconditionally negative.

Subsequently, we consider the derivatives of the macroscopic rate constants ρ_i,

$$\begin{aligned}\rho_1'(h) &= \frac{\alpha_1 e_2 - (k_{-1}+k_2)(\alpha_1 e_2/d_r + \alpha_1)}{k_1(1 + h\alpha_1 e_2/d_r + \alpha_1 h)^2},\\ \rho_2'(h) &= \frac{\alpha_2 e_4 - (k_{-3}+k_4)(\alpha_3 e_4/d_r + \alpha_2)}{k_3(1 + h\alpha_2 e_4/d_r + \alpha_2 h)^2}.\end{aligned}$$

The above derivatives may be positive for appropriately chosen parameter values when $\text{inh}_{\text{tot},0} \approx 0$. In fact, when $e_2 >> e_1 > e_{-1}, e_4 >> e_3 > e_{-3}$, and $\text{inh}_{\text{tot},0} \approx 0$, it follows that $\alpha_1 e_2 \approx e_1, \alpha_2 e_4 \approx e_3, \alpha_1 \approx 0, \alpha_2 \approx 0$, which means that

$$\begin{aligned}\rho_1 &\approx \frac{k_{-1}+k_2+e_1\text{inh}_{\text{tot},0}}{k_1(1+\frac{e_1\text{inh}_{\text{tot},0}}{d_r})},\\ \rho_2 &\approx \frac{k_{-3}+k_4+e_3\text{inh}_{\text{tot},0}}{k_3(1+\frac{e_3\text{inh}_{\text{tot},0}}{d_r})},\\ \rho_1' &\approx \frac{e_1(1-\frac{k_{-1}+k_2}{d_r})}{k_1(1+\frac{e_1\text{inh}_{\text{tot},0}}{d_r})^2},\\ \rho_2' &\approx \frac{e_3(1-\frac{k_{-3}+k_4}{d_r})}{k_3(1+\frac{e_3\text{inh}_{\text{tot},0}}{d_r})^2}.\end{aligned}$$

These estimates imply in turn that

$$\begin{aligned}\frac{\rho_1'}{\rho_1^2} &\approx \frac{e_1 k_1(1-\frac{k_{-1}+k_2}{d_r})}{(k_{-1}+k_2+e_1\text{inh}_{\text{tot},0})^2},\\ \frac{\rho_2'}{\rho_2^2} &\approx \frac{e_3 k_3(1-\frac{k_{-3}+k_4}{d_r})}{(k_{-3}+k_4+e_3\text{inh}_{\text{tot},0})^2}.\end{aligned} \tag{10}$$

When $k_{-1}+k_2 < d_r, k_{-3}+k_4 < d_r$, Equations (10) imply that both $\frac{\rho_1'}{\rho_1^2}, \frac{\rho_2'}{\rho_2^2} > 0$, and the derivative M' can be positive at $\text{inh}_{\text{tot},0} \approx 0$. Thus, for appropriately chosen rate constants k_i, e_i and such that

- $e_2 >> e_1, e_4 >> e_3$,
- d_r is large and $d_r/d_f >> 1$,

under small increments of the parameter $\text{inh}_{\text{tot}} > 0$, the steady state value $\hat{x}_3 = [\text{C}_{\text{PP}}]$ in equation (8) is monotone increasing in inh_{tot} in some neighbourhood of 0. This means that the dose response curve of $[\text{C}_{\text{PP}}]$ vs. inh_{tot} will be upward sloping in some neighbourhood of $\text{inh}_{\text{tot}} = 0$, and the motif exhibits hormesis.

For $e_2 = e_4 = 0$ (which is often assumed in the literature), $\rho_1', K_3' < 0$ unconditionally, so $M' < 0$ unconditionally, and the steady state value $\hat{x}_3 = [\text{C}_{\text{PP}}]$ in Equation (8) is monotone decreasing in inh_{tot}. Therefore, $e_2, e_4 > 0$, together with $d_r/d_f >> 1$ are necessary conditions for hormesis to occur. However, the above argument does not imply that this condition is sufficient, that means that dose response curve of C_{PP} vs. inh_{tot} may not be upward sloping in a neighbourhood of 0 for *any* $e_2, e_4 > 0$.

Note that the above argument was developed under the simplifying assumption that $e_{-2} = e_{-4} = 0$. However, a numerical study illustrates that our system exhibits hormesis even when $e_{-2}, e_{-4} > 0$ (Fig. A1) and for a range of values of $e_{-2}, e_{-4} > 0$ the dose response curve of C_{PP} vs. inh_{tot} remains upward sloping. In fact, for small $\text{inh}_{\text{tot}} > 0$ the difference between the curves is insignificant.

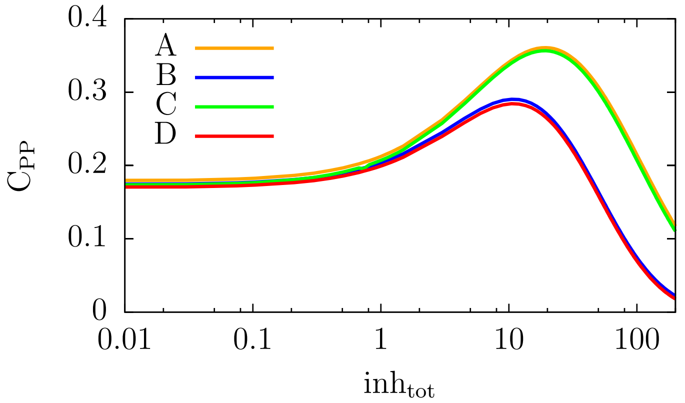

Figure A1. **The hormetic effect exists also for positive e_{-2}, e_{-4}.** Dose response curves (log scale x-axis) are compared for several combination pairs e_{-2}, e_{-4}: A. $e_{-2} = e_{-4} = 0$, B. $e_{-2} = e_{-4} = 500$, C. $e_{-2} = 0, e_{-4} = 500$, D. $e_{-2} = 500, e_{-4} = 0$. Model parameters listed in Tables A1 and A2, and $S_{tot} = 8.44$.

3. Bifurcation Diagrams

A bifurcation analysis is performed for (5) when no inhibitor is present, i.e. $\text{inh}_{\text{tot}} = 0$. To study the range of bistability of the motif output (double phosphorylated substrate, C_{PP}) the total substrate mass S_{tot} is varied. Fig. A2 shows that the steady state value $[C_{PP}]$ is hormetic in response to the total substrate mass S_{tot}. In particular, low concentrations of S_{tot} stimulate C_{PP} while high concentrations inhibit it. However, we stress that a hormetic response of C_{PP} to an inhibitor observed in Fig. 1 of the main text, does not occur due to the presence of an underlying hormetic response of C_{PP} to S_{tot}. This is ensured by carrying out numerical simulations presented in Fig.1 of the main text for sufficiently high value of S_{tot} (namely $S_{tot} = 8.44$) in whose neighbourhood the plotted curve C_{PP} vs. S_{tot} is downward sloping (Fig. A2).

The dose response is computed via continuation of a solution shown on Fig. A2 (where $\text{inh}_{\text{tot}} = 0$) by varying the total amount of inhibitor inh_{tot}.

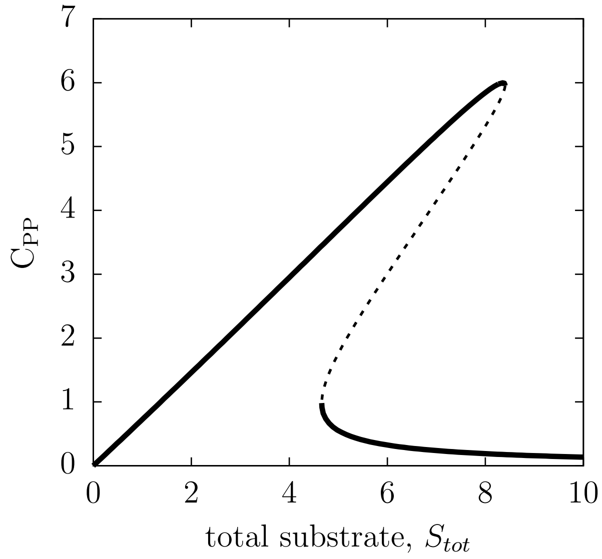

FIGURE A2. **The dual phoshorylation-dephosphorylation motif exhibits bistability without inhibition.** A plot of pairs $(S_{tot}, \mathrm{C_{PP}})$ demonstrates this fact which is well known from the theoretical literature [1, 5, 6, 7, 10]. The bistability range is $S_{tot} \in (4.660, 8.401)$, and the dashed curves represent unstable steady states. Model parameters listed in Tables A1 and A2.

4. Dose response curves

Figs. 1 and 2 of the main text illustrate the hormetic dose responses of $\mathrm{C_{PP}}$ to an inhibitor when system (5) exhibits a mono- (Fig. 1) or bi-stable (Fig. 2) behaviour in the absence of an inhibitor. For the sake of completeness, we include dose response curves for nine out of thirteen state variables in (5) for the monostable (Fig. A3) and bistable (Fig. A4) case. For the reasoning behind the examples refer to Fig. 1, 2 in the Main Text. The steady state values plotted in all dose response curves are computed via a continuation algorithm for the solutions of the nonlinear system on the right-hand side of the ODE system (5). Solution continuation and bifurcation analysis of the ODE system is performed numerically by Matcont [2, 3].

5. Logistic fit to data

The logistic function of the form

$$f(x) = \frac{a_{\max} - a_{\min}}{1 + (x/IC_{50})^{\alpha}} + a_{\min},$$

was fitted to sample data shown in Fig.3 of the Main Text using NonLinearModel.fit routine in MATLAB. The best fit parameters values are presented in Table A3.

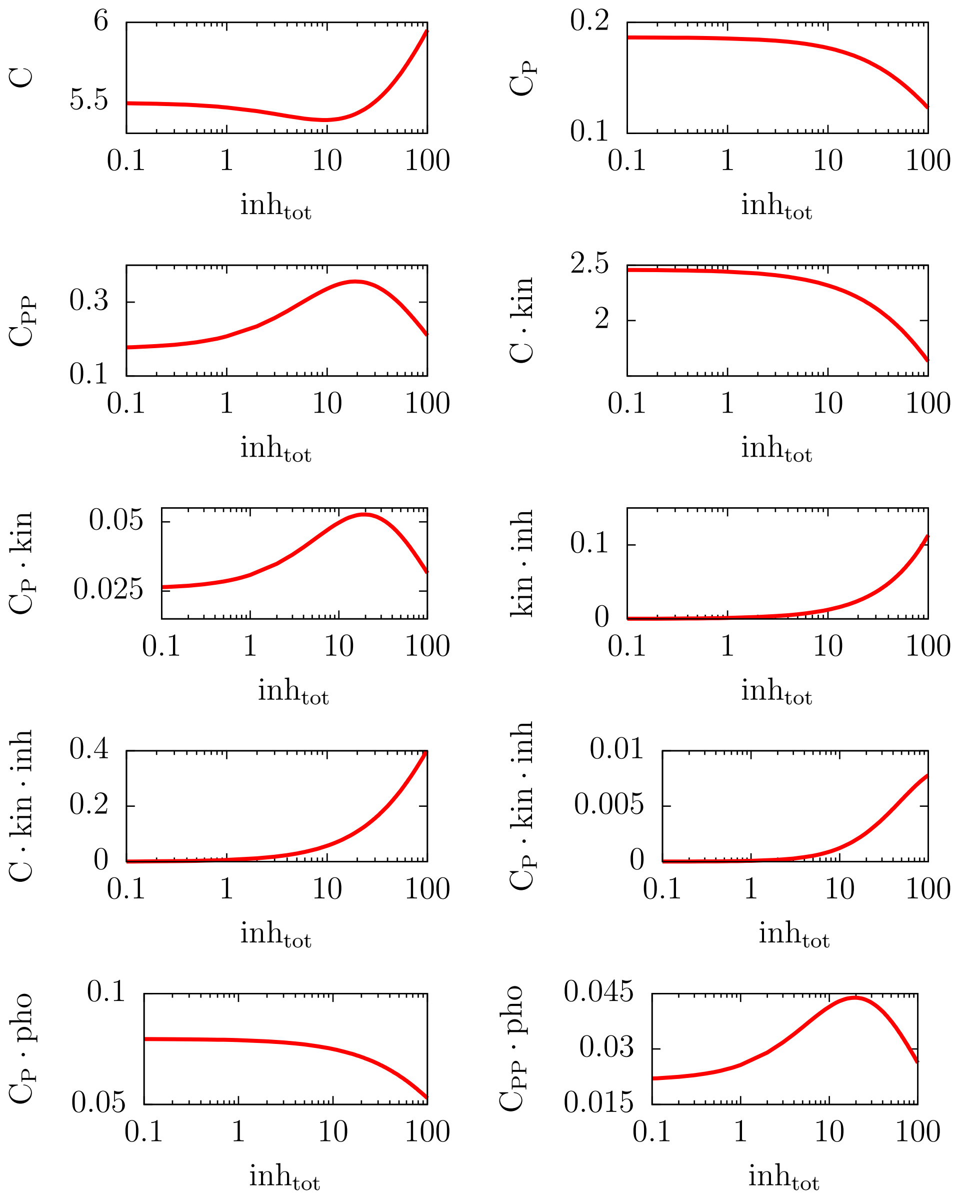

FIGURE A3. **Monostable motif. Dose response curves (log scale x-axis) for C, C_P, C_{PP}, C·kin, C_P·kin, kin·inh, C·kin·inh,C_P·kin·inh, C_P·pho, C_{PP}·pho.** Total mass of substrate used in the simulation $S_{tot} = 8.44$. Rate parameters in Tables A1 and A2.

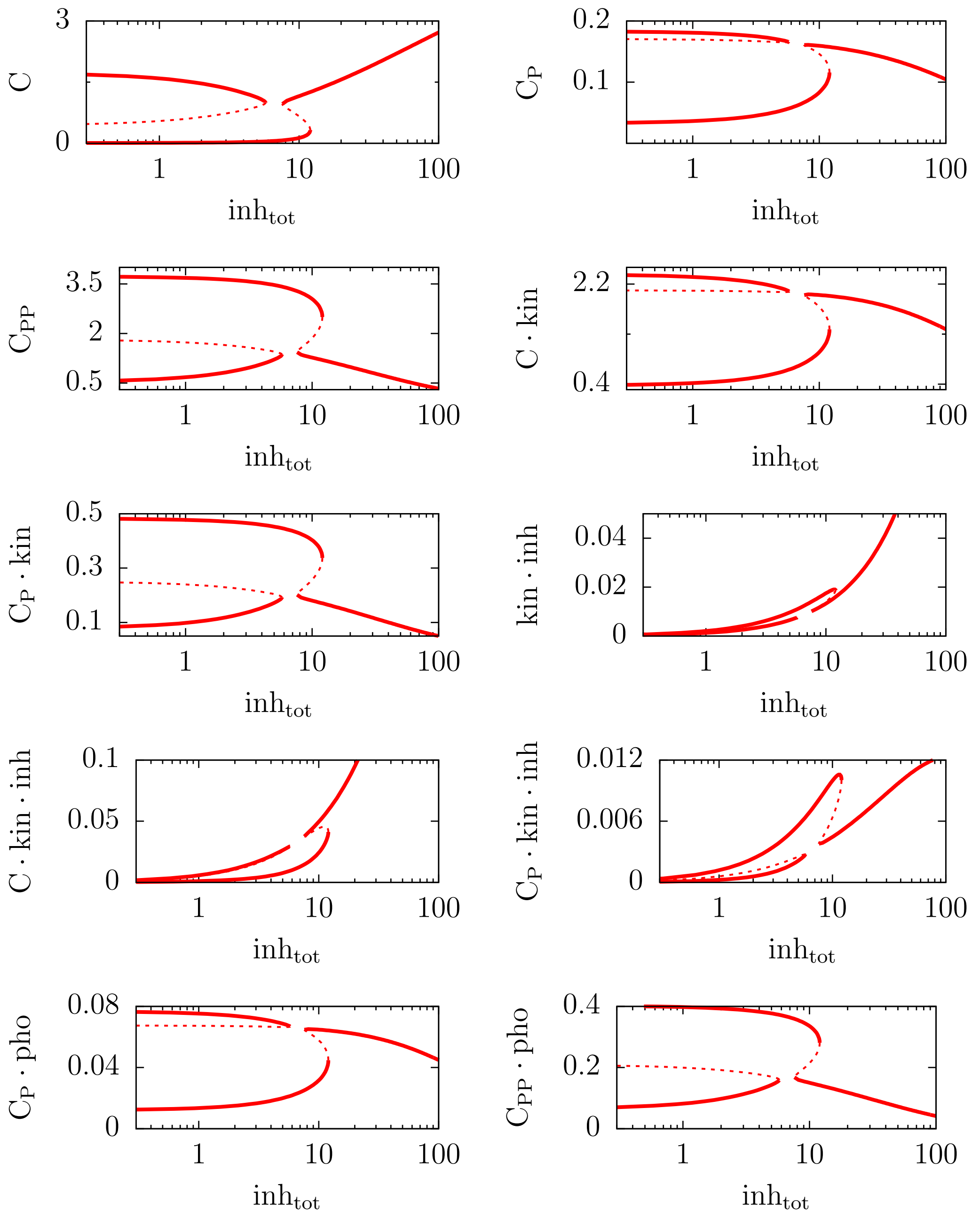

FIGURE A4. **Bistable motif. Dose response curves (log scale x-axis) for C, C_P, C_{PP}, C·kin, C_P·kin, kin·inh, C·kin·inh,C_P·kin·inh, C_P·pho, C_{PP}·pho.** Total mass of substrate used in the simulation S_{tot} = 5.04. Rate parameters in Tables A1 and A2. The dashed curves represent unstable steady states.

6. Model parameters

Table A1. **Total masses used in numerical simulations.**

Quantity	Compound	Value
S_{tot}	substrate	8.44 (Fig. 1 in the Main Text)
S_{tot}	substrate	5.04 (Fig. 2 in the Main Text)
S_{tot}	substrate, type-1 cell	4.65 (Fig. 3 in the Main Text)
S_{tot}	substrate, type-2 cell	5.04 (Fig. 3 in the Main Text)
K_{tot}	kinase	2.50
P_{tot}	phosphatase	2.50

Table A2. **Reaction rates used in numerical simulations.**

Rate	Reaction	Value	Source
k_1	C + kin → C · kin	45	estimate
k_{-1}	C · kin → C + kin	1.00	estimate
k_2	C · kin → C_P + kin	0.55	estimate
k_3	C_P + kin → C_P · kin	45	estimate
k_{-3}	C_P · kin → C_P + kin	1.00	estimate
k_4	C_P · kin → C_{PP} + kin	4.00	estimate
k_5	C_{PP} + pho → C_{PP} · pho	0.30	estimate
k_{-5}	C_{PP} · pho → C_{PP} + pho	1.00	estimate
k_6	C_{PP} · pho → C_P + pho	4.80	estimate
k_7	C_P + pho → C_P · pho	3.20	estimate
k_{-7}	C_P · pho → C_P + pho	1.00	estimate
k_8	C_P · pho → C + pho	17.00	estimate
d_f	kin + inh → kin · inh	1	d_r/d_f comparable to [9]
d_r	kin · inh → kin + inh	1000	d_r/d_f comparable to [9]
e_1	C · kin + inh → C · kin · inh	0.50	estimate
e_{-1}	C · kin · inh → C · kin + inh	1.00	estimate
e_2	C · kin · inh → C + kin · inh	200	estimate
e_{-2}	C + kin · inh → C · kin · inh		as shown
e_3	C_P · kin + inh → C_P · kin · inh	0.50	estimate
e_{-3}	C_P · kin · inh → C_P · kin · inh	1.00	estimate
e_4	C_P · kin · inh → C_P + kin · inh	200	estimate
e_{-4}	C_P + kin · inh → C_P · kin · inh		as shown

Table A3. Parameters and p-values for logistic fit.

Parameter	Value	p-value
IC_{50}	18.477	$3.3587 \cdot 10^{-6}$
$a_{\max}$	3.0768	$7.8629 \cdot 10^{-9}$
$a_{\min}$	0.43524	$1.0333 \cdot 10^{-3}$
α	3.5631	$2.0328 \cdot 10^{-4}$

7. Robustness

As observed in Fig. 5 of the Main Text, the population IC_{50} values falls in the region where the type-1 subpopulation C_{PP} decreases, while type-2 C_{PP} increases. A numerical simulation has been performed to check the robustness of this observation against parameter variation. The substrate mass S_{tot} has been varied within the bistability range (between 4.66 and 8.4) alongside the second-order constant k_3 (between 25 and 65) and the observation remained robust. However, the magnitude of the hormetic responses of the type-2 subpopulation at the IC_{50} value is sensitive to the changes in those parameters.

Consider the example in Fig. A5. In the absence of the inhibitor there are two stable steady states of the double-phosphorylated substrate (A) ${C_{PP,1}}^*$ and (B) ${C_{PP,2}}^*$. Cell populations at these two steady state will react differently to the presence of an inhibitor: (A) type-1 cells at ${C_{PP,1}}^*$ will in general exhibit an inhibitory dose-response while (B) cells at ${C_{PP,2}}^*$ will exhibit a hormetic dose response for sufficiently low inhibitor doses. At the population IC_{50} value, both populations have the same steady-state value ${C_{PP,1/2}}^*$. Yet, the type-2's hormetic response at this value is 168%, much lower than in Fig. 5 in the Main Text.

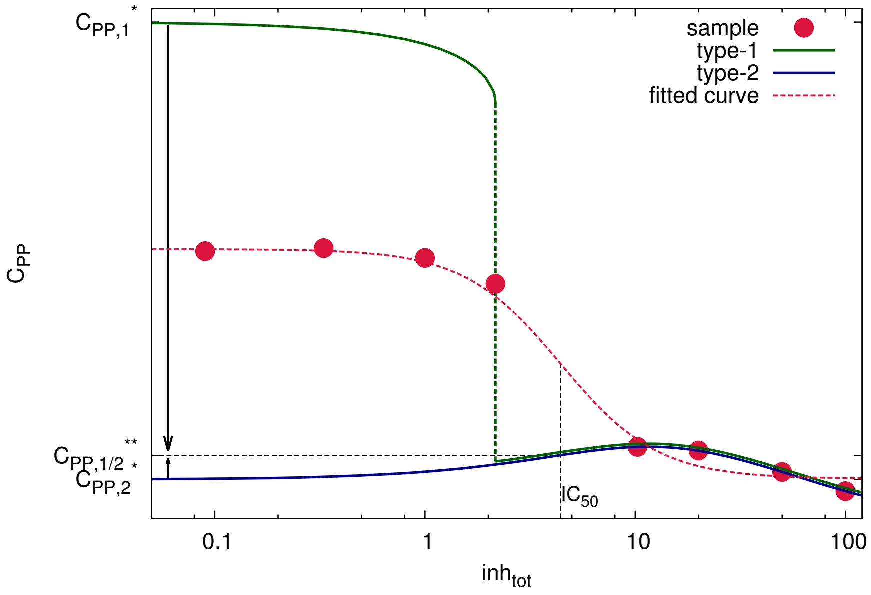

Parameter	Value	p-value
IC_{50}	4.4239	0.0421
$a_{\max}$	1.796	$8.7286 \cdot 10^{-5}$
$a_{\min}$	0.2650	0.0253
α	1.8855	0.0439

Figure A5. Dose response curve (log scale) for the double-phosphorylated substrate C_{PP} in the presence of an inhibitor (inh_{tot}), for a bistable motif. Type-1 and type-2 subpopulations are both at 50%. The dotted lines indicate a discontinuous jump in the steady state values of C_{PP} in the presence of the inhibitor. The parameter values are those shown in Table A1 except that $S_{tot} = 5.1, k_3 = 25$.

References

[1] Conradi C, Mincheva M (2014) Catalytic constants enable the emergence of bistability in dual phosphorylation. J R Soc Interface 11: 20140158.

[2] Dhooge A, Govaerts W, Kuznetsov YA (2003) MATCONT: A MATLAB package for numerical bifurcation analysis of ODEs. ACM Trans Math Software 29: 141–164.

[3] De Witte V, Govaerts W, Kuznetsov YA, Friedman M (2012) Interactive initialization and continuation of homoclinic and heteroclinic orbits in MATLAB. ACM Trans Math Software 38: 1–34.

[4] Huang CY, Ferrell JE (1996) Ultrasensitivity in the mitogen-activated protein kinase cascade. Proc Natl Acad Sci USA 93: 10078–10083.

[5] Markevich NI, Hoek JB, Kholodenko BN (2004) Signaling switches and bistability arising from multisite phosphorylation in protein kinase cascades. J Cell Biol 164: 353–359.

[6] Ortega F, Garcés JL, Mas F, Kholodenko BN, Cascante M (2006) Bistability from double phosphorylation in signal transduction. FEBS Journal 273: 3915–3926.

[7] Qiao L, Nachbar RB, Kevrekidis I, Shvartsman SY (2007) Bistability and oscillations in the Huang-Ferrell model of MAPK Signaling. PLoS Comput Biol 3: e184.

[8] Schoeberl B, Eichler-Jonsson C, Gilles ED, Müller G (2002) Computational modeling of the dynamics of the MAP kinase cascade activated by surface and internalized EGF receptors. Nat. Biotechnol 20: 370–375.

[9] Sturm OE, Orton R, Grindlay J, Birtwistle M, Vyshemirsky V, Gilbert D, Calder M, Pitt A, Kholodenko B, Kolch W (2010) The mammalian MAPK/ERK pathway exhibits properties of a negative feedback amplifier. Sci Signal 3: ra90.

[10] Wang L, Sontag ED (2008) On the number of steady states in a multiple futile cycle. J Math Biol 57: 29–52.
